# Point-of-care ultrasound for undifferentiated and musculoskeletal presentations in UK primary care: a scoping review protocol

**DOI:** 10.3399/BJGPO.2025.0189

**Published:** 2026-06-17

**Authors:** Peter A Brookes, Carolyn A Chew-Graham, Aaron Poppleton

**Affiliations:** 1 Orthopaedic and Rehabilitation Centre, University Hospitals Birmingham NHS Foundation Trust, Birmingham, UK; 2 School of Medicine, Keele University, Keele, UK

**Keywords:** ultrasonography, point-of-care, ultrasonography, primary health care, diagnosis

## Abstract

**Background:**

Point-of-care ultrasound (PoCUS) is increasingly used internationally yet remains emergent in UK primary care. While momentum is growing, variation in application and infrastructure persists. Undifferentiated and musculoskeletal (MSK) symptoms, common and diagnostically complex in general practice, may particularly benefit from PoCUS. International evidence suggests PoCUS improves diagnostic accuracy, reduces referrals to specialist care and enhances patient satisfaction. However, UK-specific evidence on PoCUS use, clinician training, governance, and implementation is sparse. With national priorities shifting towards community-based, technology-enabled care, understanding current PoCUS use is increasingly important.

**Aim:**

To systematically map the current use of PoCUS in UK primary care for undifferentiated and MSK presentations, identifying clinical applications, user profiles, implementation contexts, and gaps in the literature.

**Design & setting:**

A scoping review of peer-reviewed and grey literature pertaining to UK primary and community care settings, including general practice, urgent care, community diagnostic hubs, and related contexts.

**Method:**

Following the JBI (formerly known as Joanna Briggs Institute) framework and Preferred Reporting Items for Systematic reviews and Meta-Analyses extension for Scoping Reviews (PRISMA-ScR) guidelines, comprehensive searches will be conducted across MEDLINE, Embase, CINAHL, Web of Science, and relevant grey literature sources. Eligible studies include those involving healthcare professionals using PoCUS in adult UK primary care. Data will be charted, synthesised descriptively and thematically, and findings presented narratively with visual mapping.

**Conclusion:**

This review will provide a comprehensive overview of PoCUS use in UK primary care, highlighting how and where it is applied, by whom, and to what effect. The findings will offer practical insights to support implementation and shape future research and service development.

## How this fits in

Point-of-care ultrasound is widely used internationally, yet its integration and application within primary care in the UK remains variable and emergent. Undifferentiated and musculoskeletal presentations, common and diagnostically complex in UK primary care, may benefit from PoCUS, with international evidence demonstrating improvements in diagnostic accuracy and patient outcomes. However, UK-specific evidence is lacking, and variations in training, governance, and implementation pose risks to patient safety and consistency of care. This scoping review addresses a critical gap by mapping current PoCUS use in UK primary care to support safe, equitable adoption and guide future research, in line with the NHS *10 Year Health Plan for England* for community-based, technologically enabled care.

## Introduction

Point-of-care ultrasound (PoCUS) refers to real-time ultrasonography used by the treating clinician to answer focused clinical questions, such as detecting deep vein thrombosis or guiding joint injections. Its portability, non-invasive nature, and rapid deployment (typically under 5 minutes) make it suitable for use within routine primary care consultations.^1^

In UK primary care, PoCUS could provide immediate access to specialist-level diagnostic information at the first point of contact. By supporting timely, accurate diagnosis, it may improve patient outcomes, optimise care pathways, and reduce healthcare inequalities.^2,3^ Additionally, PoCUS may reduce unnecessary referrals, investigations, and waiting times, contributing to more efficient, patient-centred care.^4–6^ For patients and families, this means fewer appointments, shorter waits, and more timely decisions. Patients consistently report high satisfaction with PoCUS, highlighting reduced anxiety and uncertainty, alongside improved communication and better understanding,^7^ factors that may encourage informed self-management.^8^

While PoCUS is well established in hospital settings,^1^ its use in UK primary care remains limited. In contrast, GPs in countries such as Germany, Denmark, and France have integrated PoCUS into routine practice, supported by formal training and national guidance.^9^ This presents an opportunity for sustainable UK implementation.

Despite growing interest, UK-specific evidence remains limited. A systematic review highlighted the paucity of NHS-specific evidence.^10^ While international studies suggest that PoCUS in primary care can change diagnoses in up to 75% of cases, alter management in 51%, and reduce referrals by 10–13%,^11^ it remains unclear whether similar impacts are being achieved, or even targeted, in the UK context. A Delphi study of ultrasound-proficient GPs from four Scandinavian countries (*n* = 45) identified MSK, abdominal, obstetric, cardiovascular, and respiratory applications as areas of greatest benefit.^11^ These align with common UK presentations to general practice, particularly undifferentiated and MSK complaints.

Undifferentiated presentations, characterised by non-specific symptoms involving multiple organ systems, are frequent and diagnostically complex. They often lead to broad investigations or specialist care referrals.^6,11^ PoCUS can provide immediate diagnostic insight to support timely, evidence-based decision-making.^1^ MSK complaints account for about 20% of primary care attendances and contribute substantially to disability and healthcare utilisation.^12^ These commonly require imaging to confirm diagnoses such as effusions or soft tissue injuries.^13^ Radiology-dependent pathways can cause delays and increase costs. PoCUS offers a rapid, cost-effective alternative, with potential to streamline care and reduce secondary care reliance.^1^

Together, undifferentiated and MSK presentations represent high-volume, high-impact challenges in UK primary care. Their relevance aligns with the NHS *10 Year Health Plan for England,* shifting care into community settings and leveraging technology and access for underserved groups.^14,15^ PoCUS may reduce avoidable admissions by enabling timely diagnostics in rural or socioeconomically disadvantaged areas.

However, barriers to safe and sustainable implementation remain. These include variability in clinician training and expertise, time constraints, medico-legal concerns, and equipment costs.^1–3^ As PoCUS use grows, these challenges are increasingly pressing. The World Organization of Family Doctors (WONCA) endorses PoCUS for appropriate indications and has called for further research into its safe integration into generalist settings.^16^ Reflecting this momentum, the Royal College of General Practitioners (RCGP) recognised PoCUS as a special interest area in November 2024.^17^

PoCUS use in UK primary care is increasing, but without national guidance, regulatory oversight, or standardised training requirements, uptake is often informal and uneven.^9^ This raises concerns about practice variation, patient safety, and equitable access. Without better understanding of how PoCUS is used, by whom, and in which contexts, the UK risks variable implementation that could limit effectiveness or compromise care quality.

To address this need for better understanding, this scoping review will systematically map existing evidence on PoCUS use for undifferentiated and MSK presentations in UK primary care. Scoping review methodology is well suited to emerging fields with fragmented evidence, allowing inclusion of diverse study types and grey literature to map key concepts, gaps, and implementation patterns to guide future policy, education, and research.^18,19^

### Review aim and questions

This scoping review aims to examine how PoCUS is currently used in UK primary care for undifferentiated and MSK presentations. It will examine clinical applications, user profiles, implementation contexts, and gaps in the literature. The review will address the following questions:

1. What are the clinical uses of PoCUS for undifferentiated and MSK presentations in UK primary care?
2. What is the professional background of PoCUS users?
3. How is PoCUS being implemented (for example, locations, protocols, pathways, equipment) in UK primary care?
4. What are the reported benefits, limitations, and challenges of using PoCUS for these presentations in UK primary care?
5. What gaps exist in the current literature regarding the use of PoCUS for undifferentiated and MSK presentations in UK primary care?

## Method

This scoping review protocol was developed in accordance with the *JBI Manual for Evidence Synthesis*.^19^ The proposed review will follow the methodological framework proposed by Arksey and O’Malley,^18^ enhanced by Levac *et al* ^20^ and reported in accordance with the PRISMA-ScR (Preferred Reporting Items for Systematic reviews and Meta-Analyses extension for Scoping Reviews) guidelines.^21^

### Eligibility criteria

Scoping review eligibility criteria were developed using the Population, Concept, and Context (PCC) framework, in accordance with JBI guidelines.^19^

#### Population

This review will include studies involving UK-based healthcare professionals working in primary care or community settings. These may include GPs and first-contact practitioners such as physiotherapists, paramedics, nurses, and midwives. Only studies involving populations aged ≥18 years will be considered; those focused solely on paediatric populations (aged <18 years) will be excluded.

#### Concept

The central concept is the clinical use of PoCUS by healthcare professionals in the assessment, diagnosis, or management of patients. This includes focused PoCUS use in undifferentiated or multi-system presentations, such as respiratory, cardiovascular, abdominal, and obstetric or gynaecological conditions; as well as MSK presentations including joint effusions, soft tissue injuries; and ultrasound-guided procedures such as joint injections or vascular access. Therapeutic ultrasound is excluded.

#### Context

Eligible studies must be set within UK-based primary or community healthcare environments. This includes general practice (in-hours and out-of-hours care), urgent care centres, home-based care, community diagnostic hubs, paramedic-led services, and other relevant community-based contexts such as care homes, prisons, and military health care. Hospital-based studies will only be included if they describe care delivered by primary or community professionals. Only studies published in English will be included. No restrictions will be placed on publication date, to allow for a comprehensive understanding of PoCUS use over time.

#### Types of evidence sources

This review will include diverse evidence sources in line with JBI methodology, reflecting the emerging, practice-driven nature of PoCUS in UK primary care. Both peer-reviewed and grey literature will be considered.

Eligible sources will include primary research (quantitative, qualitative, and mixed-methods studies) alongside non-research sources such as clinical audits, case reports, service evaluations, quality improvement projects, and local or national guidelines. Grey literature will include policy and strategic documents (for example, from NHS England, Health Education England, and the Centre for Advanced Practice), as well as reports from professional bodies such as the RCGP and the Chartered Society of Physiotherapy. Relevant theses, dissertations, and conference abstracts will also be included, provided sufficient methodological or contextual detail is available.

### Search strategy

The search strategy was developed in collaboration with an academic librarian at Keele University and guided by Peters *et al*.^19^ Searches will be conducted across major databases including MEDLINE, Embase, CINAHL, and Web of Science. A draft Embase strategy is provided in Supplementary information 1, with alternative database searches tailored appropriately.

Grey literature will be identified through targeted searches of OpenGrey, OpenDOAR, EThOS (British Library), BASE (Bielefeld Academic Search Engine), and relevant institutional and organisational repositories. Manual searches using single-string and advanced search operators will follow key terms derived from the PCC framework. Additional sources will include studies known to the review team and identified through personal communication, including contact with study authors, subject experts, and researchers working on unpublished or in-progress material. Reference lists of all included studies will also be screened to capture further eligible sources.

The complete and reproducible search strategy will be reported in the final scoping review.

### Evidence screening and selection

Following the search, all identified citations will be uploaded into Mendeley (version 2.135.0) for removal of duplicates before transferring to Rayyan^22^ for screening. Following an initial pilot, titles and abstract screening will be conducted by one reviewer, with a second reviewer independently verifying a sample (20%) to ensure consistency and accuracy. Discrepancies will be resolved through discussion, with a third reviewer consulted if needed. Full-text screening will be undertaken by one reviewer and verified by a second. Reasons for exclusion at full-text stage will be recorded. The process will be documented using a PRISMA-ScR flow diagram.^21^

### Data charting

A standardised data charting tool was developed collaboratively by the review team (Supplementary information 2), adapted from the JBI data extraction tool to reflect the review aims. The form will be pilot tested on a small sample of studies. Any modifications will be documented.

Data charting will be undertaken by one reviewer and verified by a second. Disagreements will be resolved through discussion or, if necessary, by consulting a third reviewer. Where data are missing, authors will be contacted (two attempts over 3 weeks). Any additional data will be documented in the data charting sheet, with source and date of receipt. CADIMA (version 2.2.4.2) will support data charting and synthesis^23^ Single-reviewer screening and charting with verification follows JBI guidance and ensures feasibility and rigour.^19^

### Data analysis and presentation

A PRISMA-ScR flow diagram will summarise the search strategy. Extracted data will be collated and synthesised using descriptive numerical summary and thematic analysis. Quantitative data, such as study characteristics, clinical applications, and user demographics, will be summarised using tables and charts. Qualitative data on implementation, benefits, challenges, and gaps will be analysed thematically using framework analysis,^24^ enabling coding against pre-defined themes while remaining open to emergent concepts. Findings will be presented narratively, with visual mapping where appropriate, to provide a comprehensive overview of PoCUS use in UK primary and community care.

### Consultation and collaboration

The multidisciplinary review team comprises two academic GPs and a physiotherapist. This collaborative approach aims to enhance the review’s relevance, rigour, and impact. The review concept and aim were developed in partnership with public collaborators within Keele University’s Public and Communities Group; this partnership will continue throughout the study. Key stakeholders, including clinicians, educators, and service managers experienced in PoCUS within UK primary care^19^ will be consulted to refine interpretation of findings, identify additional literature, and inform recommendations for practice and future research.

## Discussion

### Summary

This scoping review will systematically map the use of PoCUS for undifferentiated and MSK presentations in UK primary care. It will clarify how PoCUS is applied, by whom, and in which clinical contexts.

### Strengths and limitations

This protocol follows Joanna Briggs Institute methodology and is informed by PRISMA-ScR guidance, supporting transparency and reproducibility. A comprehensive search across multiple databases, together with inclusion of diverse study designs, will enable a broad mapping of the evidence on PoCUS use in UK primary care.

As a scoping review, no formal quality appraisal of included studies will be undertaken, limiting assessment of the robustness of the evidence base. The breadth of inclusion criteria may introduce heterogeneity across studies, and restriction to English-language publications may result in language bias and omission of relevant evidence.

### Implications for research and practice

By synthesising diverse sources, this review will highlight current practice patterns, identify barriers and facilitators to use, and expose areas where further research is required. Findings will be of relevance to clinicians, educators, commissioners, and policymakers amid growing national interest. It will address an important evidence gap and provide foundational insights to support safe, effective, and equitable implementation across community-based settings.

Ultimately, this work seeks to ensure that the potential benefits of PoCUS are realised in ways that are clinically appropriate, evidence-informed, and aligned with NHS priorities for community-based, patient-centred care.

## Trial registration number

This scoping review protocol is registered with the Open Science Framework: https://doi.org/10.17605/OSF.IO/4CKHX.

## Data

No primary data are reported in this protocol. Data generated from the scoping review, including extracted study data and data charting forms, will be available from the corresponding author on reasonable request.
